# eEF1A1 binds and enriches protoporphyrin IX in cancer cells in 5-aminolevulinic acid based photodynamic therapy

**DOI:** 10.1038/srep25353

**Published:** 2016-05-06

**Authors:** Zhichao Fan, Xiaojun Cui, Dan Wei, Wei Liu, Buhong Li, Hao He, Huamao Ye, Naishuo Zhu, Xunbin Wei

**Affiliations:** 1 State Key Laboratory of Oncogenes and Related Genes, Shanghai Cancer Institute, School of Biomedical Engineering, Shanghai Jiao Tong University, Shanghai, China; 2 Institutes of Biomedical Sciences, Fudan University, Shanghai, China; 3 Laboratory of Molecular Immunology, State Key Laboratory of Genetic Engineering, School of Life Sciences, Fudan University, Shanghai, China; 4 Key Laboratory of Optoelectronic Science and Technology for Medicine of Ministry of Education, Fujian Provincial Key Laboratory for Photonics Technology, Fujian Normal University, Fuzhou, Fujian, China; 5 Department of Urology, Changhai Hospital, Second Military University, Shanghai, China; 6 Cell Death and Survival Networks Program, Sanford Burnham Prebys Medical Discovery Institute, La Jolla, California, USA; 7 Division of Inflammation Biology, La Jolla Institute for Allergy & Immunology, La Jolla, California, USA

## Abstract

Photodynamic therapy (PDT) with protoporphyrin IX (PpIX), which is endogenously derived from 5-aminolevulinic acid (5-ALA) or its derivatives, is a promising modality for the treatment of both pre-malignant and malignant lesions. However, the mechanisms of how ALA-induced PpIX selectively accumulated in the tumors are not fully elucidated. Here we discovered that eukaryotic translation elongation factor 1 alpha 1 (eEF1A1) interacted with PpIX (with an affinity constant of 2.96 × 10^6^ M^−1^). Microscopy imaging showed that ALA-induced PpIX was co-localized with eEF1A1 in cancer cells. eEF1A1 was found to enrich ALA-induced PpIX in cells by competitively blocking the downstream bioavailability of PpIX. Taken together, our study discovered eEF1A1 as a novel photosensitizer binding protein, which may play an essential role in the enrichment of ALA-induced PpIX in cancer cells during PDT. These suggested eEF1A1 as a molecular marker to predict the selectivity and efficiency of 5-ALA based PDT in cancer therapy.

Photodynamic therapy (PDT) is increasingly being recognized as an attractive and alternative treatment modality for cancers^1^,^2^,^3^,^4^,^5^. 5-aminolevulinic acid (5-ALA), as the precursor of protoporphyrin IX (PpIX) in heme biosynthesis pathway^6^, is one of the most important reagents used in cancer PDT of the prostate^7^, breast 8, skin^9^,^10^, cervix^11^, bladder^12^, brain^13^,^14^, lung^15^, stomach^16^, head and neck^17^, and colon^18^, as well as those for leukemia^19^ and other types of cancer.

Many studies have aimed on the mechanism of 5-ALA-induced PpIX selective accumulation in tumor lesion. One hypothesis focuses on the target proteins which can interact with PpIX: the peripheral benzodiazepine receptor (PBR)^19^, p53^18^, fragile histidine triad protein (FHIT)^20^ or low density lipoprotein (LDL) has been proposed as target proteins. Some of these proteins, such as LDL, can be enriched from blood into tumor cells compared to normal tissues^21^. Other studies have shown positive or negative correlations between PpIX accumulation and peptide transporter 1 (PEPT1)^12^ or ATP-binding cassette sub-family G member 2 (ABCG2)^12^,^13^ expressions, respectively. However, some of the studies above are inconsistent. For example, p53 and FHIT are demonstrated as tumor suppressors, which are rarely expressed in cancer cells. Therefore, the mechanism of how 5-ALA-induced PpIX accumulated selectively in tumor lesions is unclear and remain further investigated.

Using the method of complementary DNA (cDNA) phage displayed library screening^22^,^23^,^24^,^25^, we discovered a novel PpIX binding protein - eukaryotic translation elongation factor 1 alpha 1 (eEF1A1), which is highly expressed in cancer cells^26^. Moreover, we measured the affinity and studied subcellular localization of eEF1A1 and PpIX. Overexpression of eEF1A1 was used to assay its function in the accumulations of 5-ALA-induced PpIX in cancer cells. In addition, we studied the mechanism of tumor-selective PpIX accumulation after exogenous intake of 5-ALA.

## Results

### eEF1A1 was identified as the novel target binding protein for PpIX

**Figure 1:**
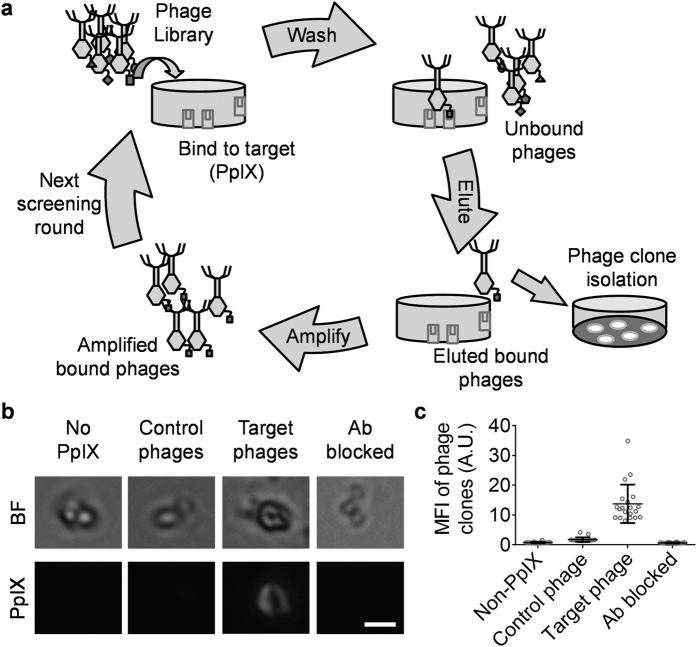
Screening of PpIX affinitive protein by T7Select Human Liver cDNA Library. (**a**) The cartoon diagrammatic drawing shows the processes of screening. (**b**) Typical fluorescence microscopy images verify the PpIX binding specificity of the protein expressed on selected phage clone. Selected phage clone shows significant PpIX binding (red) compared to no-PpIX-added and non-specific phage clones. eEF1A1 antibody blockade eliminate the PpIX binding. Scale bar is 10 μm. (**c**) The PpIX MFI of n = 20 phage clones in each data set. Mean ± SD.

To investigate the mechanism of PpIX accumulation in PDT targeted cells, we sought to find out the PpIX-binding protein(s). Using phage displayed cDNA library technology^22^,^23^,^24^,^25^, we obtained the phage clone with the strongest and most selective PpIX-binding ability after four rounds of screening (Fig. 1a). Fluorescence microscopy imaging showed (Fig. 1b,c) that our selected clone has significant PpIX binding, which had considerable PpIX florescence intensity (mean florescence intensity, MFI = 13.75 ± 6.43 arbitrary units, A.U., n = 20), compared to the clone of PpIX non-specific phages (MFI = 1.75 ± 0.75 A.U., n = 20).

To identify the peptide displayed on selected phage surface, the cDNA inserted into the phages were amplified by polymerase chain reaction (PCR) and sent for sequencing. After sequence alignment in GeneBank by basic local alignment search tool (BLAST), we found out that the peptide expressed on the selected phage (IKAVDKKAAGAGKVTKSAQKAQKAK) was the C terminal lysine motif of eEF1A1 and rich in basic amino acids. Blocking the peptides by an anti-eEF1A1 C terminal polyclonal antibody eliminated the PpIX-binding to the selected phage clones (Fig. 1b,c, MFI = 0.62 ± 0.18 A.U., n = 20). Therefore, we have identified eEF1A1 as a PpIX-binding protein.

### eEF1A1 interacts with ALA-induced PpIX

**Figure 2:**
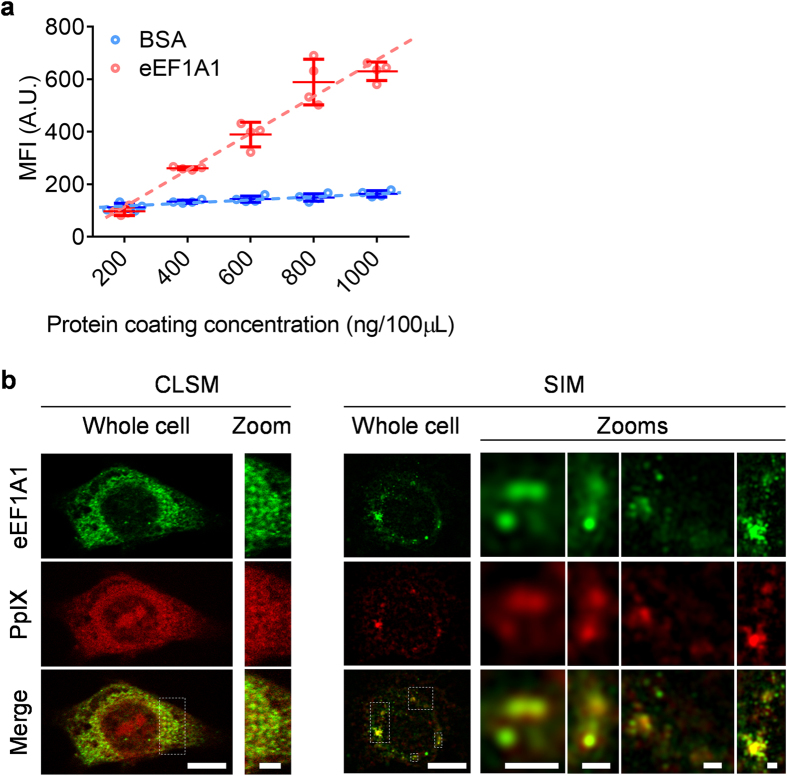
*In vitro* analysis of the interaction between eEF1A1 and PpIX. (**a**) Correlations of coated purified eEF1A1 protein and BSA (control) or binding PpIX (fluorescence intensity). n = 4 samples per data set; Mean ± SD. (**b**) The representative images present the co-localization of FITC-labeled eEF1A1 (green) and PpIX (red) *in situ* acquired by both CLSM and SIM. Areas highlighted by white dot frames in whole cell images are zoomed to show details. Scale bars for whole cell images are 10 μm. Scale bar for zoomed CLSM image is 3 μm. Scale bars for zoomed SIM images are 1 μm.

To evaluate the binding between eEF1A1 and PpIX, we first measured the fluorescence of PpIX bond to immobilized eEF1A1 protein. Compared to bovine serum albumin (BSA) control, eEF1A1 presented significant higher binding of PpIX (Fig. 2a), which was quantified by MFI. A linear correlation was observed between MFI of bound PpIX and amount of coated eEF1A1 (R^2^ = 0.9736). In BSA control, the increase of bond PpIX was much less noticeable, which represented the non-specific binding of PpIX. A linear correlation in control group was also observed (R^2^ = 0.9571), whereas ~11.7 times lower slope was shown compared to eEF1A1 group. Significant differences were present when comparing the regression lines of two groups (P < 0.0001) using F-test and comparing the data sets of two groups in each concentration points (400–100 ng/100 μl, P < 0.01) using t-test.

Surface plasmon resonance (SPR) assay was performed to quantify the affinity constant (association constant, K_a_) of eEF1A1-PpIX binding. A K_a_ of 2.96 × 10^6^ M^−1^was obtained in eEF1A1–PpIXbinding, which was significantly higher than the K_a_ of PpIX-BSA (~1.8–2.3 × 10^5^ M^−1^, ref. 37)^27^, PpIX-HSA (Human Serum Albumin, ~1.5–2.1 × 10^5^ M^−1^)^27^, or PpIX-IgG (immunoglobulin G, ~1.9–2.4 × 10^5^ M^−1^)^27^, and even higher than some antibody-antigen binding^28^.

Immunofluorescence staining of 5-ALA incubated cells was utilized to investigate whether this eEF1A1-PpIX interaction was present in cells. Using confocal laser scanning microscopy (CLSM) and structured illumination microscopy (SIM)^29^, co-localizations of PpIX and eEF1A1 were observed in HepG2 cells after 24-hour incubation with 5-ALA (Fig. 2b). Most PpIX was co-localized with eEF1A1 protein in the cytoplasm, except for a small portion of PpIX located in the nucleus.

To further validate the co-localization of PpIX and eEF1A, co-dependence was obtained by evaluating Pearson’s correlation coefficient^30^ for PpXI and eEF1A1 staining. A coefficient of 0.58 ± 0.09 (n = 5) indicated a significant correlation between PpXI and FITC-labeled anti-eEF1A1-antibody pixel intensity profiles. This was further supported by larger Manders’ overlap coefficients of 0.84 ± 0.03 (n = 5) and co-localization coefficient of 0.92 ± 0.09 and 0.96 ± 0.07 (n = 5) for M1 (indicating the co-localization of PpXI with anti-eEF1A1-FITC) and M2 (indicating the co-localization of anti-eEF1A1-FITC with PpXI), respectively^30^. The above analytical procedures avoid cursory assignment of PpXI and eEF1A1 co-localization merely by inspection.

We further performed intensity correlation analysis (ICA)^31^, which is another important objective measure of co-dependent staining between a pair of fluorescence channels and involves computation of intensity changes of corresponding pixels in each channel. The resulting intensity correlation quotient (ICQ) was 0.22 ± 0.02 (n = 5), which indicated that PpXI and eEF1A1 were co-localized and varied synchronically.

### eEF1A1 promotes the accumulation of ALA-induced PpIX in cells

**Figure 3:**
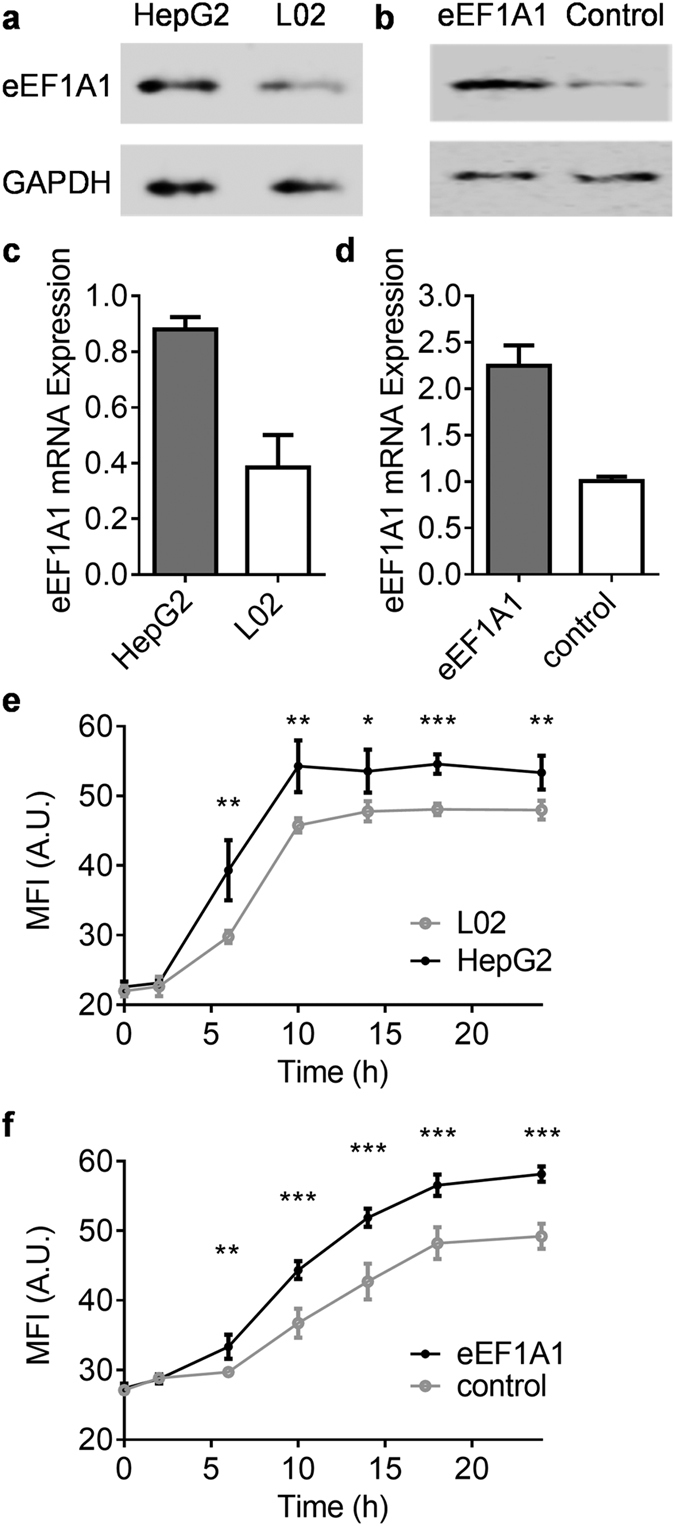
PpIX accumulation in living cells correlated to the expression of eEF1A1. (**a–d**) The expression of eEF1A1 in HepG2 and L02 cells (**a**,**c**) or HepG2 cells transfected by eEF1A1 reconstructed or control plasmids (**b**,**d**) assessed by western blot (**a**,**b**) and qRT-PCR (**c**,**d**). (**e,f**) Time-lapse measurements of ALA-induced PpIX accumulations in HepG2 and L02 cells (**e**) or HepG2 cells transfected by eEF1A1 reconstructed or control plasmids (**f**). Mean ± SD, n = 4 samples per data set, *p < 0.05, **p < 0.01, ***p < 0.001.

To investigate the function of the strong interaction between eEF1A1 and PpIX, we studied the relationship between eEF1A1 expression and accumulation of the ALA-induced PpIX in cells. Hepatocellular carcinoma cells are known to express higher eEF1A1 than normal hepatocytes^32^. Indeed, more eEF1A1 were expressed in HepG2 cancer cell line than L02 noncancerous hepatic cell line, which was assessed by both quantitative reverse transcription-polymerase chain reaction (qRT-PCR, Fig. 3a) and western blotting (Fig. 3c). Correspondently, by measuring PpIX fluorescent intensity, we found out that more ALA-induced PpIX accumulated in HepG2 cells than L02 cells (Fig. 3e). Specifically, the ALA-induced PpIX increased dramatically after the addition of 5-ALA in HepG2 cells and reached the saturation at about hour 10. In contrast, the ALA-induced PpIX in L02 cells increased slower than that in HepG2 cells and reached to the peak at about hour 14. Significant differences between the two groups started to manifest from hour six.

To demonstrate whether this increasing enrichment was related to eEF1A1, we overly expressed eEF1A1 protein in HepG2 cells. Compared to HepG2 transfected with empty plasmid, HepG2 transfected with eEF1A1-reconstituted plasmid produced higher eEF1A1 messenger RNA (mRNA, Fig. 3b) and expressed higher amount of eEF1A1 protein (Fig. 3d). Interestingly, eEF1A1 over-expressed HepG2 showed higher amount of ALA-induced PpIX accumulation than control HepG2 (Fig. 3f). Significant differences between the two groups were observed from hour 5.

### eEF1A1 prevents PpIX degradation

**Figure 4:**
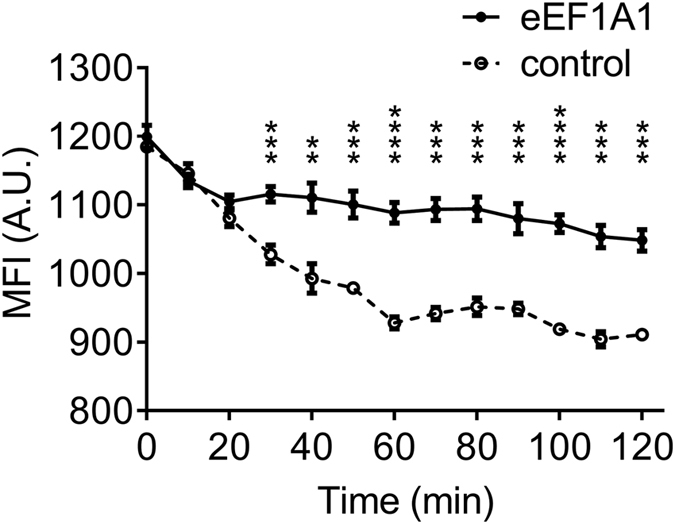
PpIX degradation in cell lysates was inhibited by eEF1A1. Time-lapse monitoring of PpIX MFI in lysates with or without adding eEF1A1 protein. PpIX is degraded significantly slower when eEF1A1 is presentcompared to that in the control. Mean ± SD, n = 3 samples per data set, **p < 0.01, ***p < 0.001, ****p < 0.0001.

We demonstrated that eEF1A1 interacted with ALA-induced PpIX and promoted accumulation of ALA-induced PpIX in cells. We hypothesized that binding to eEF1A1 would prevent PpIX bioavailability in the downstream metabolism. By measuring PpIX degradation in HepG2 cell lysate, we found out that adding purified eEF1A1 protein significantly inhibited PpIX degradation compared to vehicle control (Fig. 4). Significant differences between the two groups were observed from minute 30.

## Discussion

Our study has identified eEF1A1 as a novel binding protein for photosensitizer ALA-induced PpIX. eEF1A1 was further confirmed to have a very strong binding affinity to PpIX, which is close to or exceeds the affinity between antigen and antibody (10^5^~10^7^ M^−1^)^28^. Moreover, our study has demonstrated that the eEF1A1-PpIX interactions were present in cancer cells and led to the accumulation of ALA-induced PpIX by preventing PpIX degradation. Since the tumorogenic potential of eEF1A1 has been proposed following the observation that its over-expression correlates with increased metastatic potential in mammary adenocarcinoma^26^,^32^,^33^, including melanomas, hepatocellular carcinoma, pancreatic carcinoma, breast carcinoma, prostatic carcinoma, cervical carcinoma and bladder carcinoma, some of which were commonly treated by PDT clinically, our finding provide an mechanistic explanation that why PpIX accumulated in cancer cells in 5-ALA based PDT.

On the other hand, eEF1A1 might serve as an target for 5-ALA based PDT. The eEF1A, including eEF1A1 and eEF1A2, are the second most abundant protein (1–3% of total protein content) after actin and an important component of translation machinery^34^. Canonical function of eEF1A1 serves as a translation factor, which delivers the aminoacylated- transfer-RNA to its binding site of the ribosome for decoding of mRNA by codon–anticodon interactions^34^ and plays key roles in protein synthesis^26^. Its protein sequence is highly conserved among eukaryotic species ranging from yeast to human. In addition to its critical role in protein biosynthesis, eEF1A1 has been implicated in broadly uncanonical function, including actin-binding and bundling^35^, apoptosis^36^, nuclear transport^37^, heat shock response^37^, cell anoikis^38^ and proteasomal-mediated degradation of damaged proteins^39^. eEF1A1 is required for normal cell growth, proliferation, and cell cycle progression^40^. Indeed, eEF1A1 was reported as a target for anti-tumor therapy^41^. Targeting eEF1A1 can combat apoptosis-resistant cancers and melanomas^36^. During 5-ALA based PDT, absorption of photon energy by the photosensitizer (PpIX) contained in tumor tissue induces an oxygen dependent photochemical reaction that most probably results in the generation of toxic reactive singlet oxygen species (ROSs)^42^. The oxidative damageoccurs in close proximity to the site where ROSs formed. Since we have demonstrated that eEF1A1 binds to photosensitizer PpIX, ROS generated during PDT will deactive eEF1A1 and eliminate its functions, which may lead to cell death. Moreover, eEF1A1 protein shares 75% nucleotide and 96% amino acid homology with its isoform eEF1A2 protein, which is also reported as diagnostic marker for cancers^26^,^43^. This suggests eEF1A2 as another potential target for 5-ALA mediated PDT.

In conclusion, this study shows the intracellular interaction between ALA-induced PpIX and eEF1A1. Furthermore, the accumulation of PpIX is correlated with eEF1A1 expression, which has provided new insights in both the mechanism of PpIX accumulation in cancer cells in 5-ALA based PDT as well as how the PDT works (target). Our study has suggested eEF1A1 expresson as a diagnosis parameter for determining therapeutic strategies, since 5-ALA based PDT might be specifically effective in treating patients who have high-expression of eEF1A1.

## Methods

### Phage displayed cDNA library screen

Phage displayed cDNA library technology is a well-designed and high-throughput protein screening method based on expressing recombinant random peptides on the surface of filamentous phage and screening functional peptides, such as RNA-binding proteins^22^, drug targeted proteins^23^, protein targeting peptides^24^ and cell targeting peptides^25^. To screen the PPIX-affinitive protein, we used the high-throughput T7 select human liver cDNA Library (Merck, Darmstadt, Germany) screening. In this library, peptides transcribed from human liver cDNA were exposed in the surface of T7 phages. To keep a high vitality, the library was amplified in host bacteria BLT5403 (Merck) to reach a titer of ~10^10^ PFU/ml for the screening.

In screening, the 96-well plate was coated with PpIX (1 μg/ml, 200 μl/well, Frontier Scientific, Logan, UT, USA) in darkness at 4 °C overnight. The 1:100 diluted amplified phages (200 °l/well) were introduced into the plate and incubated at 37 °C for 30 min with gentle rocking. After five washes with phosphate buffered saline (PBS), BLT5403 were added into the wells to harvest the target phages. The eluted BLT5403 containing target phages were amplified and subjected to additional rounds of selection. The titers of phages in each round were monitored to assess the enrichment of phages during screening. Four rounds of the affinity selection process were performed (Fig. 1a) to screen out PpIX binding proteins.

### Cloning of the phages presenting PpIX binding proteins

The screened out phages were plated at low density (less than 100 PFU/plate). The plaques were transferred onto nitrocellulose (NC) filter membrane (Schleicher &Schuell, Dassel, Germany) *in situ* at 37 °C for 1 h. After three washes with PBS, NC membrane was incubated in 1ug/ml PpIX with gentle shaking at 37 °C for 30 min and imaged by epifluorescence microscope (Olympus IX51, Olympus Corp., Tokyo, Japan, Ex: 530 nm / Em: 630 nm). The plaque with highest PpIX fluorescence was cloned from the plate.

To verify the PpIX binding of selected phage clone, lysate smear (including lysogenic bacteria and phage) of selected phage clone and control (no PpIX binding) were incubated with or without 1 μg/ml PpIX at 37 °C for 30 min and imaged by fluorescence microscope. In some experiments, rabbit anti-human eEF1A1 polyclonal antibody (10 ug/ml, 37 °C, 10 min, Santa Cruz Biotech Inc., Santa Cruz, CA, USA) was used to block PpIX binding of selected phage clone. The mean florescence intensity (MFI) of phage clones in fluorescence images were analyzed by “analyze particles” in FIJI-ImageJ2^44^.

### Identification of protein presented by selected phage clone

Encoding sequences of PpIX binding peptide in selected phage clone were amplified by PCR with the phage lysate as the template. The primers used in PCR were designed as T7 UP Primer 5′-AACCCCTCAAGACCCGTTTA-3′ and T7 DOWN primer 5′-GGAGCTGTCGTATTCCAGTC-3′. The PCR products were analyzed by agarose electrophoresis. The inserted gene was sequenced by Invitrogen (Shanghai, China). Homology analysis between obtained sequences and human genes listed in GeneBank (National Center for Biotechnology Information) were performed by using BLAST.

### Plasmid construction

cDNA of human eEF1A1 gene were amplified from total mRNA of human fetal liver (BiocolorBioScience& Technology, Shanghai, China) by reverse transcription-polymerase chain reaction (RT-PCR). The primers used in PCR were designed as 5′-CTGAATTCATGGGAAAGGAAAAGACT-3′ and 5′-ACTGGATCCTTTAGCCTTCTGAGCTTTCTG-3′. The eEF1A1 cDNA were cloned into pEGFP-C1 (BD Clontech, Palo Alto, CA) and verified by PCR, restriction enzyme digestion and DNA sequencing (Invitrogen).

### Cell culture and transfection

HepG2 cells and L02 cells were purchased from Cell Bank of Shanghai Institute of Biochemistry and Cell Biology (Chinese Academy of Sciences, Shanghai, China). Cells were used in related experiments within 6 month after received the cells from the bank. The cells were performed short tandem repeat (STR) and mycoplasma tests in the bank. Cells were cultured in RPMI 1640 complete medium (Gibco, Rockville, MD) containing 10% fetal bovine serum (HyClone, South Logan, UT) at 37 °C with 5% CO_2_. For transfection, empty (pEGFP-C1) or eEF1A1-reconstructed plasmids were transfected into HepG2 cells using Lipofectamine 2000 (Life Technologies, Shanghai, China) according to the manufacturer’s instructions.

### qRT-PCR

RNA in L02 cells, HepG2 cells and transfected HepG2 cells was extracted using TRIzol (Invitrogen). cDNA was synthesized using FastQuant RT Kit (with gDNase) (TIANGEN, Beijing, China) according to the manufacturer’s instructions. Expression level of eEF1A1 mRNA was measured by Roche Lightcycler 480 (Roche, Nutley, NJ, USA) using SuperRealPreMix Plus (SYBR Green, TIANGEN) and normalized to expression level of GAPDH.

### Western blot

Cell lysates of L02 cells, HepG2 cells and transfected HepG2 cells were fractionated by sodium dodecyl sulfate polyacrylamide gel electrophoresis (SDS-PAGE) and detected by immunoblotting using rabbit anti-human eEF1A1 polyclonal antibody (1:1000) and horseradish peroxidase (HRP)-conjugated goat anti-rabbit antibody (1:5000, Santa Cruz Biotech Inc.). Sample loading amounts in the assay were normalized by GAPDH (rabbit anti-human GAPDH polyclonal antibody, 1:2000, Santa Cruz Biotech Inc.) blotting. The blots were visualized by G:BOX Gel & Blot Imaging Systems (Syngene, Cambridge, England).

### Fluorescence binding assay

The 96-well plate was coated by recombinant human eEF1A1 (Abnova, Taipei, Taiwan) or control BSA (Sigma, Fremont, CA, USA) with a series of concentrations (200-1000 ng/100 μl) at 4 °C overnight. After three washes with PBS, PpIX (1 μg/ml, 200 μl/well) were added into the plate and incubated at 37 °C for 1.5 h. After three washes with PBS, the fluorescence of PpIX was measured in SpectraMax M5 microplate reader (Molecular Devices Inc., Sunnyvale, CA, USA). The excitation and emission wavelength during the measurements are 400 and 620 nm, respectively.

### SPR assay

Label-free SPR based technology^45^ for studying biomolecular interactions in real time was used to assess the K_a_ of eEF1A1-PpIX interaction. SPR measurements were conducted following manufacturer’s protocol. Briefly, the eEF1A1 as ligand was immobilized to a CM5 sensory chip via amine coupling. A reference chamber was processed by ethanolamine. Various concentrations of PpIX were injected into the chip. The response curves, calculated as the difference between responses in the sample chamber and the reference chamber, were recorded to assess the binding. The sensor chip was regenerated with 2 molar NaCl between measurements. The sensorgrams were fitted using a one-site binding model.

### Immunofluorescence

To monitor the intracellular localizations of eEF1A1 and ALA-induced PpIX, HepG2 cells were cultured with medium containing 5-ALA (1 mM, Sigma) for 24 hours and fixed by 4% paraformaldehyde for 10 min at room temperature (RT). The cells were permeabilized by 0.5% Triton X-100 (Sigma) for 1 h at RT and treated with blocking buffer containing 2% BSA, 10% goat serum (Gibco) and 0.1% Tween-200 (Sigma) for 1 h. The samples were incubated with rabbit anti-human eEF1A1 polyclonal antibody (10 μg/ml) at 4 °C overnight and incubated with fluorescein isothiocyanate (FITC)-conjugated goat anti-rabbit antibody (10 μg/ml, Boster Biotechnology Corp., Wuhan, China) at 37 °C for 30 min. All the operations were protected from light.

### Microscopy

Fluorescence image of eEF1A1 and ALA-induced PpIX in immunofluorescence samples were acquired by a CLSM (Leica TCS SP5, Leica Microsystems CMS GmbH, Am Friendenplatz, Mannheim, Germany) using a 40× oil-immersion objective (NA = 1.25) as described before^46^,^47^,^48^,^49^. FITC-labeled eEF1A1 was excited by 488 nm argon laser and detected by a photomultiplier tube (PMT) with the bandpass filter of 500–550 nm. ALA-induced PpIX was co-excited by 405 nm and 561 nm lasers (around the two peaks of PpIX excitation) to achieve maximum excitation and detected by a PMT with the bandpass filter of 590–650 nm.

Intracellular localizations of eEF1A1 and ALA-induced PpIX were also assessed by super resolution SIM imaging (N-SIM microscopy system on a Ti microscope, Nikon Instruments Inc, Melville, NY) with a resolution of ~100 nm^29^. The samples were imaged with a 100X oil-immersion objective (NA = 1.49) and an Andor DU-897 EM-CCD camera (Andor Technology PLC, Belfast, Northern Ireland). A 488 nm and a 561 nm laser beam were used to excite FITC-labeled eEF1A1 and PpIX, respectively.

### Intensity correlation analysis

The intensity correlation analysis was done by evaluating Pearson’s correlation coefficient (Rr, range −0.12–1), Manders’ overlap coefficient (R, range 0–1) and co-localization coefficient (M1, M2, range 0–1)^30^ and performing intensity correlation analysis (ICA)^31^ with ImageJ plugin as described previously^30^,^31^,^50^. For R, Rr, M1 and M2, a value of 1 represents total colocalization while a value of 0 represents no colocalization. For ICA, the output - intensity correlation quotient (ICQ) is from the range of −0.5–0.5. A value from 0–0.5 indicates the intensities of the two channels vary synchronically.

### Accumulation of ALA-induced PpIX

The HepG2 cells and L02 cells (8,000 cells/well) were seeded in 96-well plates and cultured for 6 hours prior to assays. During the assay, cells were incubated in serum-free RPMI-1640 medium with or without 1 mM 5-ALA in darkness. Time-lapse measurements of PpIX fluorescence were performed by SpectraMax M5 microplate reader as described above. Soluble 5-ALA or PpIX in medium were washed out before measurements so that the fluorescence only represents intercellular PpIX. In some experiments, HepG2 cells transfected with empty or eEF1A1 reconstructed plasmid were served as experimental or control groups.

### PpIX degradation assay

HepG2 cells were lysed by nondenaturing lysis buffer (Cell Signaling Technology Inc, Danvers, MA, USA) containing protease inhibitor PMSF (phenylmethylsulfonyl fluoride, 1 mM, Life Technologies) on ice for 10 min. The cell lysate was mixed with PpIX (1 μg/ml) and eEF1A1 (1.3 μg/ml, Abnova). The lysate mixed with PpIX was used as a control group. The lysate mixed with vehicles or eEF1A1 were used as blank controls for normalization. Time-lapse measurements of PpIX fluorescence were performed by SpectraMax M5 microplate reader as described above. The lysate were incubated at 37 °C between the measurements.

### Statistics

Statistical analysis was performed with Prism 6 (GraphPad). Data were presented as mean ± standard deviation (SD). Single data points were presented in some graphs. The means for the data sets were compared, each separately, using student t-tests with equal variances. Linear regressions were performed for some data sets. The slopes of the linear regressions for the data sets were compared using an F-test. P values less than 0.05 were considered significant.

## Additional Information

**How to cite this article**: Fan, Z. *et al.* eEF1A1 binds and enriches protoporphyrin IX in cancer cells in 5-aminolevulinic acid based photodynamic therapy. *Sci. Rep.* **6**, 25353; doi: 10.1038/srep25353 (2016).
